# Cyclin B1: A potential prognostic and immunological biomarker in pan-cancer

**DOI:** 10.17305/bb.2024.10253

**Published:** 2024-10-01

**Authors:** Quan Chen, Li Ouyang, Qing Liu

**Affiliations:** 1 Department of Pathology, Hubei Provincial Hospital of Traditional Chinese Medicine, Wuhan, China; 2 Hospital Department, Hubei University of Chinese Medicine, Wuhan, China

**Keywords:** Cyclin B1 (CCNB1), pan-cancer, prognosis, immunization, biomarker

## Abstract

Cyclin B1 (*CCNB1*) encodes a regulatory protein essential for the regulation of cell mitosis, particularly in controlling the G2/M transition phase of the cell cycle. Current research has implicated *CCNB1* in the progression of various types of cancer, including gastric cancer, breast cancer, and non-small cell lung cancer. In this study, we conducted a pan-cancer analysis of *CCNB1* to investigate its prognostic significance and immunological aspects. Our comprehensive investigation covered a wide range of analyses, including gene expression, promoter methylation, genetic alterations, immune infiltration, immune regulators, and enrichment studies. We utilized multiple databases and tools for this purpose, such as The Cancer Genome Atlas (TCGA), the Genotype-Tissue Expression (GTEx) project, the Human Protein Atlas (HPA), the University of Alabama at Birmingham CANcer data analysis Portal (UALCAN), the Gene Expression Profiling Interactive Analysis (GEPIA), the DNA Methylation Interactive Visualization Database (DNMIVD), the Search Tool for the Retrieval of Interacting Genes/Proteins (STRING), Sangerbox, and cBioPortal. Data analyses were executed using GraphPad Prism software, R software, and various online tools. Our findings demonstrated a significant increase in *CCNB1* expression across 28 cancer types. Elevated *CCNB1* expression correlated with decreased overall survival (OS) in 11 cancer types and disease-free survival (DFS) in 12 cancer types. Additionally, DNA promoter methylation levels were significantly decreased in 14 cancer types. Furthermore, the study verified a significant association between *CCNB1* expression and immune infiltration, immune modulators, microsatellite instability (MSI), and tumor mutational burden (TMB). Enrichment analysis indicated that *CCNB1* is involved in multiple cellular pathways. Collectively, our results suggested that *CCNB1* has the potential to serve as a valuable prognostic biomarker and may be a promising target for immunotherapy in various cancer types.

## Introduction

Cancer is a highly concerning global disease that significantly impacts patients’ quality of life and imposes substantial economic burdens [1]. With one in four individuals at risk of developing cancer in their lifetime, the absence of effective treatments for complete eradication is alarming [2]. Consequently, researchers worldwide are increasingly focusing on gene therapy as a promising therapeutic option to prevent cancer-related mortality [3]. It is therefore imperative to further explore the relationship between genes and carcinogenesis.

Cyclin B1 (CCNB1) is a regulatory protein that plays a crucial role in promoting cell mitosis during the G2/M phase [4]. CCNB1 forms a protein complex with the cyclin-dependent kinase 1 (CDK1) to facilitate the precise localization of the monopolar spindle 1 kinase (MPS1) at the kinetochore, ensuring the accuracy of mitosis [5]. The CCNB1/CDK1 complex regulates the transition from G2 to M phase and is essential for initiating mitosis [6, 7]. The dysregulation of CCNB1 results in uncontrolled cell growth and may contribute to the development of malignant tumors. Numerous studies have demonstrated aberrant expression of CCNB1 in various types of cancer. Recent research has shown that a higher expression of CCNB1 is linked to a poorer prognosis in certain cancers, such as gastric cancer, breast cancer, and non-small-cell lung cancer [8–10]. Additionally, CCNB1 has been implicated in the pathogenesis of various cancers [11–14]. Several recent studies have demonstrated a correlation between CCNB1 and immune infiltration [15, 16]. However, the majority of studies on CCNB1 have been limited to specific types of cancer. Therefore, our objective is to systematically characterize the role of CCNB1 in pan-cancer.

The pan-cancer analysis is crucial for elucidating the principles of carcinogenesis. The current availability of comprehensive biological data in publicly accessible databases provides a convenient avenue for conducting pan-cancer analyses. We conducted a comprehensive pan-cancer analysis to investigate the previously unexplored, crucial role of CCNB1.

## Materials and methods

### Databases

The RNA expression and clinical data were acquired from The Cancer Genome Atlas (TCGA) database (https://www.cancer.gov/ccg/research/genome-sequencing/tcga) and the Genotype-Tissue Expression (GTEx) project (https://www.genome.gov/Funded-Programs-Projects/Genotype-Tissue-Expression-Project). Both the Gene Expression Profiling Interactive Analysis (GEPIA) 2.0 (http://gepia2.cancer-pku.cn/#index) and Sangerbox (http://vip.sangerbox.com/login.html) were utilized as online analysis tools for RNA expression analysis in this manuscript, with Sangerbox additionally providing a clinico-pathological analysis model. The University of Alabama at Birmingham CANcer data analysis web Portal (UALCAN) (https://ualcan.path.uab.edu/index.html) enables comprehensive analyses utilizing data from TCGA [17]. The Human Protein Atlas (HPA) (https://www.proteinatlas.org/) database is a comprehensive effort to systematically map the complete human proteome by integrating antibody-based proteomics with various other omics techniques. UALCAN and HPA were employed for the assessment of protein expression levels across different types of cancer. The cBioPortal for Cancer Genomics (https://www.cbioportal.org/) offers a web-based platform for exploring, visualizing, and analyzing complex cancer genomics data [18]. In this study, we conducted the methylation analysis using UALCAN and performed the genetic mutation analysis through cBioPortal. The Search Tool for the Retrieval of Interacting Genes/Proteins (STRING) database (https://cn.string-db.org/) facilitated the analysis of customizable protein–protein interaction (PPI) networks in *Homo sapiens*. The interacting proteins of CCNB1 were retrieved from the STRING database. The Database for Annotation, Visualization, and Integrated Discovery (DAVID) (https://david.ncifcrf.gov/) was utilized for conducting the Kyoto Encyclopedia of Genes and Genomes (KEGG) pathway and Gene Ontology (GO) enrichment analyses.

This study encompasses 39 different types of cancer, including adrenocortical carcinoma (ACC), acute lymphoblastic leukemia (ALL), bladder urothelial carcinoma (BLCA), breast invasive carcinoma (BRCA), cervical squamous cell carcinoma (CESC), cholangiocarcinoma (CHOL), colon adenocarcinoma (COAD), colorectal adenocarcinoma (COADREAD), lymphoid neoplasm diffuse large B cell lymphoma (DLBC), esophageal carcinoma (ESCA), glioblastoma (GBM), glioma (GBMLGG), brain lower grade glioma (LGG), head and neck squamous cell carcinoma (HNSC), renal chromophobe carcinoma (KICH), renal clear cell carcinoma (KIRC), renal papillary cell carcinoma (KIRP), pan-kidney cohort (KIPAN), acute myeloid leukemia (LAML), liver hepatocellular carcinoma (LIHC), lung adenocarcinoma (LUAD), lung squamous cell carcinoma (LUSC), mesothelioma (MESO), ovarian serous cystadenocarcinoma (OV), pancreatic adenocarcinoma (PAAD), pheochromocytoma and paraganglioma (PCPG), prostate adenocarcinoma (PRAD), rectum adenocarcinoma (READ), sarcoma (SARC), skin cutaneous melanoma (SKCM), stomach adenocarcinoma (STAD), stomach and esophageal carcinoma (STES), testicular germ cell tumors (TGCT), thyroid carcinoma (THCA), thymoma (THYM), uterine corpus endometrial carcinoma (UCEC), uterine carcinosarcoma (UCS), uveal melanoma (UVM), and high-risk Wilms tumor (WT).

### Gene expression analysis

The GEPIA 2.0 database was utilized to analyze the differential messenger RNA (mRNA) expression levels of CCNB1 across various cancers and their corresponding normal tissues, integrating data from the TCGA and GTEx databases. The expression data of CCNB1 from GEPIA were visualized using a body map and a dot plot. Similarly, the Sangerbox database was utilized to conduct a comprehensive analysis of CCNB1 mRNA expression levels in different tumor tissues and their respective normal tissues. The expression data were normalized using log2 (TPM + 0.001) for the log scale. The UALCAN online tool was utilized to analyze protein expression. Furthermore, determining the expression of CCNB1 at the histological level was essential. Therefore, we downloaded immunohistochemical images of 12 tumor tissues with their corresponding normal tissues from the HPA database to evaluate the protein levels of CCNB1 across different cancer types.

### Clinico-pathological phenotype analysis

The Sangerbox database was utilized to evaluate the association between the CCNB1 expression levels and clinico-pathological parameters, including clinical stage, tumor grade, T-stage, N-stage, and M-stage, in patients with various types of cancers from the TCGA database. The samples with no detectable expression levels were removed, and cancers represented by fewer than three samples were excluded. Additionally, a log2 (*x* + 0.001) transformation was applied to each expression value. We performed logistic regression analysis to assess the clinical significance of CCNB1 by investigating its correlation with different clinical stages. The data were analyzed using GraphPad Prism version 9.5 and presented through the receiver operating characteristics (ROC) curve visualization. In this study, stages I and II were classified as early stages, while stages III and IV were classified as late stages.

### Survival analysis

We evaluated the overall survival (OS) and disease-free survival (DFS) to assess the correlation between the CCNB1 expression and patient prognosis across various types of cancer. Two statistical analyses were conducted, specifically the Cox regression analysis and the Kaplan–Meier analysis. RNA-sequencing expression profiles and corresponding clinical data from the TCGA database were obtained for the survival analysis. Cox regression analysis was conducted using the “forest plot” R package and analyzed with R software, version 4.0.3. A forest plot was used to display the *P* value, hazard ratio (HR), and the 95% confidence interval (CI) for each variable. The Kaplan–Meier analysis was conducted using the “survival analysis” module within the GEPIA database.

### DNA methylation analysis

Analysis of the methylation levels of the CCNB1 gene promoter was conducted using the UCLCAN database, based on the TCGA dataset.

### Genetic alteration analysis

We utilized data from the TCGA Pan-cancer Atlas Studies, which comprised a total of 10,967 samples, for the analysis of genetic alterations. The cBioPrortal online tool was used to analyze the frequency of alterations and the types of alterations of CCNB1 in all samples. The mRNA expression *z* scores (RNA Seq V2 RSEM) were acquired using a *z* score threshold of ± 2.0, in accordance with professional standards.

### Correlation of CCNB1 expression with immune infiltration and immune checkpoint genes

The data for 33 types of cancer and their corresponding normal tissues were obtained from the TCGA database. We utilized the “immuneeconv” R software package, which integrates the Tumor Immune Estimation Resource (TIMER) and xCELL algorithms, to calculate immune scores for this study. Transcripts associated with the immune checkpoint include sialic acid binding Ig like lectin 15 (*SIGLEC15*), indoleamine 2,3-dioxygenase 1 (*IDO1*), cluster of differentiation 274 (*CD274*), hepatitis A virus cellular receptor 2 (*HAVCR2*), programmed cell death protein 1 (*PDCD1*), cytotoxic T-lymphocyte associated protein 4 (*CTLA4*), lymphocyte activation gene 3 (*LAG3*), and programmed cell death 1 ligand 2 (*PDCD1LG2*). We analyzed the expression of these eight genes and observed the expression values related to the immune checkpoint for CCNB1. Spearman correlation analysis was performed to generate heatmaps of the relationship between *CCNB1* gene expression and genes linked to immune scores or checkpoints across various cancer types. The vertical axis of the heatmaps represents different immune scores, and the colors indicate correlation coefficients.

### Correlation of CCNB1 expression with the tumor mutational burden (TMB) and microsatellite instability (MSI)

We acquired the TMB and MSI scores from the TCGA database and performed statistical analyses using R software, including Spearman correlation analysis of TMB, MSI, and CCNB1 gene expression. The *x*-axis of the graphical representation illustrated the magnitude of correlation between CCNB1 expression and either TMB or MSI, while the *y*-axis identified different tumor types. The size of the data points reflected the correlation coefficient and varying colors denoted the significance of the *P* values, with a darker shade of blue representing smaller *P* values. Two-group comparisons were conducted using the Wilcoxon test. The data deviated from a normal distribution. *P <* 0.05 were considered statistically significant.

### Enrichment analysis of CCNB1

We obtained the PPI network for CCNB1 from the String database, limiting the number of interactors to fewer than 50 and setting the minimum required interaction score to low confidence. From this, only experimentally verified CCNB1-binding proteins were selected. The top 100 CCNB1-related target genes were identified using the “Similar Gene Detection” module of the GEPIA database. By employing a Venn diagram, we identified ten common genes across the two datasets. Pearson correlation analysis between CCNB1 and these related genes was conducted using the “Correlation Analysis” module of GEPIA. Subsequently, genes from both datasets were subjected to KEGG pathway and GO enrichment analyses. The gene list was downloaded from the DAVID database and the data was visualized using the Sangerbox web tool.

### Statistical analysis

Data are presented as means ± standard deviation (SD). Differences between normal and tumor samples were assessed using Student’s *t*-test or the Wilcoxon test, the latter being employed for data that deviated from a normal distribution. Statistical analyses were conducted using R software, version 4.0.3, and GraphPad Prism 9.5. *P* values less than 0.05 were considered statistically significant. Levels of significance were denoted as *P < 0.05, **P < 0.01, ***P < 0.001, and ****P < 0.00001.

## Results

### Pan-cancer analysis of CCNB1 mRNA and protein expression

**Figure 1.**
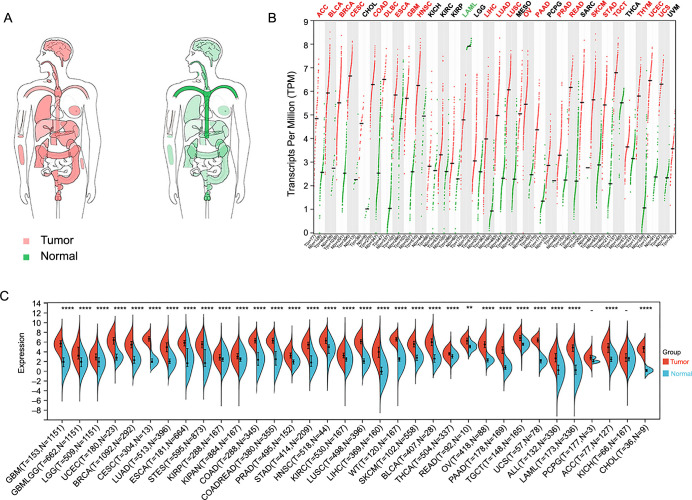
**Comprehensive expression analysis of CCNB1 across different cancer types.** (A) Body map illustrating the CCNB1 mRNA expression levels in various tumors compared with normal tissues; (B) Dot plot depicting the median mRNA expression levels of CCNB1 in tumor vs normal samples as obtained from the GEPIA database. Each dot corresponds to the expression level in a single sample; (C) Violin plots representing the mRNA expression levels of CCNB1 in 34 different tumor tissues and their corresponding normal tissues, based on the Sangerbox database. *P < 0.05; **P < 0.01; ***P < 0.001; and ****P < 0.00001. CCNB1: Cyclin B1; mRNA: Messenger RNA; GEPIA: Gene Expression Profiling Interactive Analysis; ACC: Adrenocortical carcinoma; ALL: Acute lymphoblastic leukemia; BLCA: Bladder urothelial carcinoma; BRCA: Breast invasive carcinoma; CESC: Cervical squamous cell carcinoma; CHOL: Cholangiocarcinoma; COAD: Colon adenocarcinoma; COADREAD: Colorectal adenocarcinoma; DLBC: Lymphoid neoplasm diffuse large B cell lymphoma; ESCA: Esophageal carcinoma; GBM: Glioblastoma; GBMLGG: Glioma; LGG: Brain lower grade glioma; HNSC: Head and neck squamous cell carcinoma; KICH: Renal chromophobe carcinoma; KIRC: Renal clear cell carcinoma; KIRP: Renal papillary cell carcinoma; KIPAN: Pan-kidney cohort; LAML: Acute myeloid leukemia; LIHC: Liver hepatocellular carcinoma; LUAD: Lung adenocarcinoma; LUSC: Lung squamous cell carcinoma; MESO: Mesothelioma; OV: Ovarian serous cystadenocarcinoma; PAAD: Pancreatic adenocarcinoma; PCPG: Pheochromocytoma and paraganglioma; PRAD: Prostate adenocarcinoma; READ: Rectum adenocarcinoma; SARC: Sarcoma; SKCM: Skin cutaneous melanoma; STAD: Stomach adenocarcinoma; STES: Stomach and esophageal carcinoma; TGCT: Testicular germ cell tumors; THCA: Thyroid carcinoma; THYM: Thymoma; UCEC: Uterine corpus endometrial carcinoma; UCS: Uterine carcinosarcoma; UVM: Uveal melanoma; WT: High-risk Wilms tumor.

To investigate the mRNA expression landscape of CCNB1 across various cancers, we comprehensively analyzed its mRNA levels using interactive body maps from the GEPIA database. This analysis revealed significant differences in the median expression levels of CCNB1 between most human tumor tissues and their corresponding normal tissues, particularly in the lung, blood, brain, breast, digestive organs, reproductive system, and colon (Figure 1A). Further, we examined the mRNA levels of CCNB1 across 33 cancer types compared to their matched normal tissues using the GEPIA database. The results indicated elevated median CCNB1 mRNA expression in 22 tumor types, including ACC, BLCA, BRCA, CESC, COAD, DLBC, ESCA, GBM, HNSC, LIHC, LUAD, LUSC, OV, PAAD, PRAD, READ, SKCM, STAD, TGCT, THYM, UCEC, and UCS (Figure 1B). Conversely, CCNB1 expression was decreased in LAML (Figure 1B). There were no significant differences in CCNB1 mRNA expression among CHOL, KICH, KIRC, KIRP, MESO, PCPG, SARC, THCA, and UVM (Figure 1B). Additionally, a comprehensive analysis using the Sangerbox web tool demonstrated overexpression of CCNB1 in 32 cancer types, including GBM, GBMLGG, LGG, UCEC, BRCA, CESC, LUAD, ESCA, STES, KIRP, KIPAN, COAD, COADREAD, PRAD, STAD, HNSC, KIRC, LUSC, LIHC, WT, SKCM, BLCA, THCA, READ, OV, PAAD, TGCT, UCS, ALL, LAML, ACC, and CHOL (Figure 1C). The results from both Sangerbox and the GEPIA database exhibited consistent trends in CCNB1 expression profiles across various cancer types.

**Figure 2.**
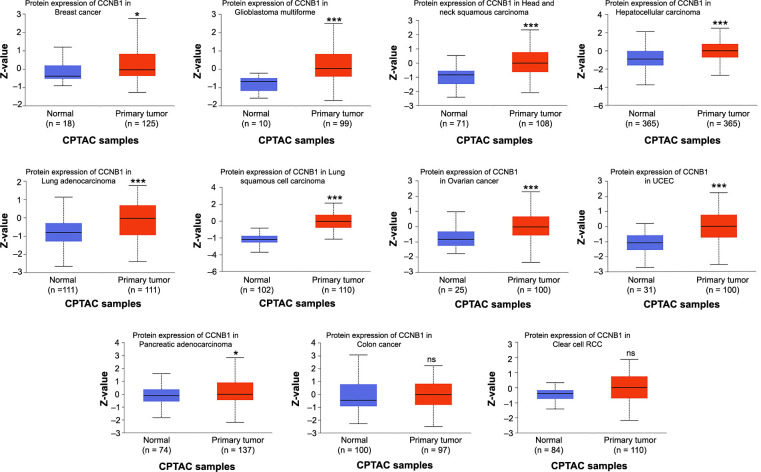
**The protein expression of the CCNB1 across various cancer types, analyzed by UALCAN.** CCNB1: Cyclin B1; UALCAN: University of Alabama at Birmingham CANcer data analysis Portal; CPTAC: Clinical Proteomic Tumor Analysis Consortium; UCEC: Uterine corpus endometrial carcinoma; RCC: Renal cell carcinoma.

Furthermore, we examined the protein levels of CCNB1 in tumors vs their corresponding normal tissues using the UALCAN database. The results revealed that total protein expression of CCNB1 was elevated in nine types of cancer, including breast cancer, GBM, HNSC, hepatocellular carcinoma, LUAD, LUSC, ovarian cancer, UCEC, and PAAD (Figure 2). The gene expression patterns were found to be consistent with their respective protein expression profiles across these cancers. However, in colon cancer and renal clear cell carcinoma, CCNB1 protein expression did not show significant differences when compared with their respective normal tissues (Figure 2).

**Figure 3.**
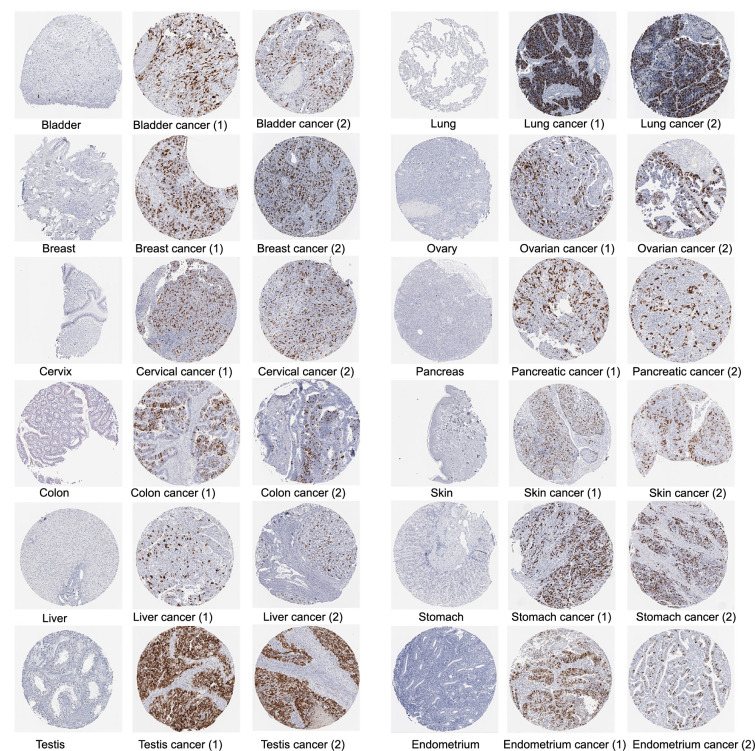
**Immunohistochemical images from the HPA database illustrating the protein expression of CCNB1 in normal tissue and various cancer types.** CCNB1: Cyclin B1; HPA: Human Protein Atlas.

We also obtained immunohistochemical images from the HPA database to assess CCNB1 protein expression in tumor samples. The immunohistochemical analysis showed elevated CCNB1 protein levels in 12 cancer types in comparison to their normal tissue counterparts, specifically in bladder, breast, cervical, colon, liver, testicular, lung, ovarian, pancreatic, skin, stomach, and endometrial cancers (Figure 3).

These findings indicate that CCNB1 is commonly upregulated across the majority of cancer types, highlighting its potential significance as a pivotal molecule in the initiation and advancement of various cancers.

### Pan-cancer analysis of the correlation between CCNB1 expression and clinico-pathological features

**Figure 4.**
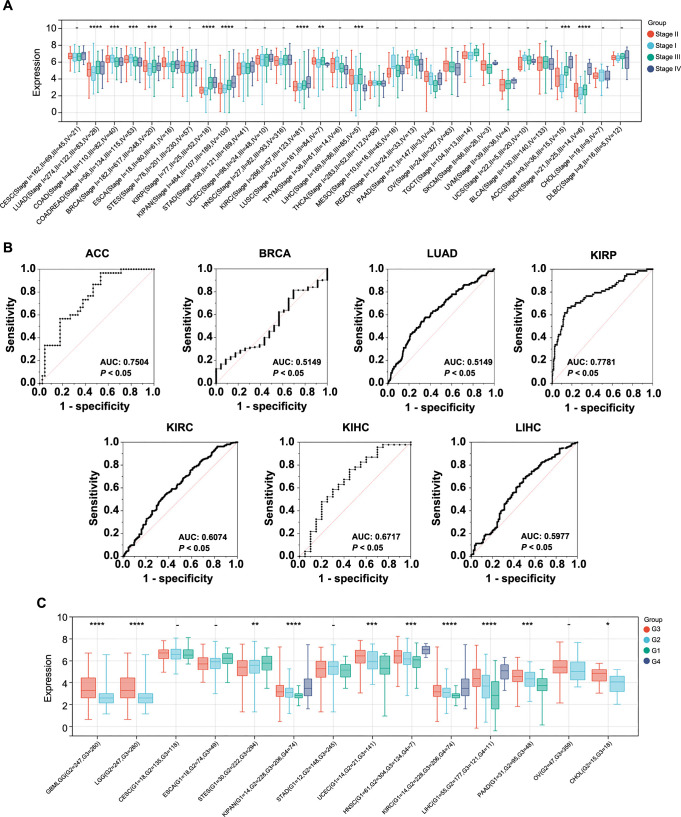
**Analysis of CCNB1 expression and its correlation with clinicopathological features in various cancers.** (A) Displaying a correlation analysis between CCNB1 expression levels with clinical stages across various cancer types; (B) Showcasing the logistic regression analysis assessing the discriminative power of CCNB1 between early and advanced clinical stages in selected cancers, with the performance measured by ROC curves; (C) Detailing the correlation between CCNB1 expression levels and histopathological grades of various cancer types. *P < 0.05; **P < 0.01; ***P < 0.001; and ****P < 0.00001. CCNB1: Cyclin B1; ROC: Receiver operating characteristics; CESC: Cervical squamous cell carcinoma; LUAD: Lung adenocarcinoma; COAD: Colon adenocarcinoma; COADREAD: Colorectal adenocarcinoma; BRCA: Breast invasive carcinoma; ESCA: Esophageal carcinoma; STES: Stomach and esophageal carcinoma; KIRP: Renal papillary cell carcinoma; KIPAN: Pan-kidney cohort; STAD: Stomach adenocarcinoma; UCEC: Uterine corpus endometrial carcinoma; HNSC: Head and neck squamous cell carcinoma; KIRC: Renal clear cell carcinoma; LUSC: Lung squamous cell carcinoma; THYM: Thymoma; LIHC: Liver hepatocellular carcinoma; THCA: Thyroid carcinoma; MESO: Mesothelioma; READ: Rectum adenocarcinoma; PAAD: Pancreatic adenocarcinoma; OV: Ovarian serous cystadenocarcinoma; TGCT: Testicular germ cell tumors; SKCM: Skin cutaneous melanoma; UVM: Uveal melanoma; UCS: Uterine carcinosarcoma; BLCA: Bladder urothelial carcinoma; ACC: Adrenocortical carcinoma; KICH: Renal chromophobe carcinoma; CHOL: Cholangiocarcinoma; DLBC: Lymphoid neoplasm diffuse large B cell lymphoma; AUC: Area under the curve; GBMLGG: Glioma; LGG: Brain lower grade glioma.

Accurate prediction of clinico-pathological parameters is crucial for tailoring treatments to individual needs and devising a rational follow-up plan to enhance patient prognosis. To assess the association between the expression of CCNB1 and clinico-pathological features in multiple cancers, we first analyzed the CCNB1 expression at different clinical stages of cancer using the Sangerbox database. Our analysis included 30 different cancers, while others were excluded due to insufficient sample sizes. The analysis revealed that the CCNB1 expression levels were significantly linked with clinical stages in 12 cancer types, namely, LUAD, COAD, COADREAD, BRCA, ESCA, KIRP, KIPAN, KIRC, LUSC, LIHC, ACC, and KICH, with an upregulation mainly noted in later stages (stages III and IV) for LUAD, BRCA, KIRP, KIRC, LIHC, ACC, and KICH (Figure 4A). To explore the potential of CCNB1 as a biomarker for distinguishing between early (stages I and II) and late (stages III and IV) stages of cancer, logistic regression analysis was performed on these seven cancer types. The ROC curves indicated significant diagnostic potential in six cancer types, which included LUAD, KIRP, KIRC, LIHC, ACC, and KICH. Notably, ACC and KIRP exhibited the area under the curve (AUC) values above 0.7, suggesting a substantial clinical significance of CCNB1 in differentiating between early and late disease stages in these cancers (Figure 4B). In other words, elevated CCNB1 expression was indicative of a worse prognosis in ACC and KIRP.

Furthermore, CCNB1 expression was found to correlate significantly with histological grades in ten cancer types, including GBMLGG, LGG, STES, KIPAN, UCEC, HNSC, KIRC, LIHC, PAAD, and CHOL. Higher expression levels of CCNB1 were observed in advanced histological grades as opposed to lower grades in GBMLGG, LGG, KIPAN, UCEC, HNSC, KIRC, LIHC, PAAD, and CHOL (Figure 4C).

**Figure 5.**
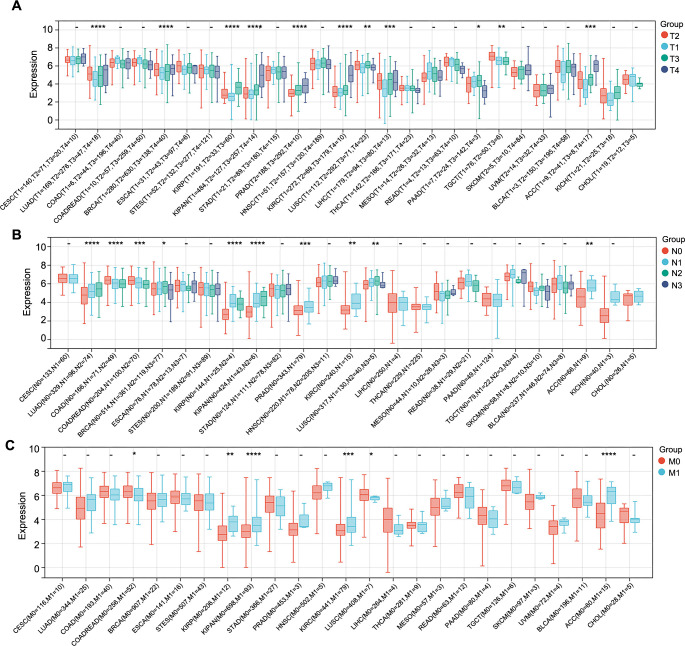
**Analysis of CCNB1 expression and its correlation with tumor stage classifications in various cancers.** (A-C) Displaying the correlation of CCNB1 expression levels with T-stages (A), N-stages (B), and M-stages (C) across various cancer types. *P < 0.05; **P < 0.01; ***P < 0.001; and ****P < 0.00001. CCNB1: Cyclin B1; T: Tumor; N: Node; M: Metastasis; CESC: Cervical squamous cell carcinoma; LUAD: Lung adenocarcinoma; COAD: Colon adenocarcinoma; COADREAD: Colorectal adenocarcinoma; BRCA: Breast invasive carcinoma; ESCA: Esophageal carcinoma; STES: Stomach and esophageal carcinoma; KIRP: Renal papillary cell carcinoma; KIPAN: Pan-kidney cohort; STAD: Stomach adenocarcinoma; PRAD: Prostate adenocarcinoma; HNSC: Head and neck squamous cell carcinoma; KIRC: Renal clear cell carcinoma; LUSC: Lung squamous cell carcinoma; LIHC: Liver hepatocellular carcinoma; THCA: Thyroid carcinoma; MESO: Mesothelioma; READ: Rectum adenocarcinoma; PAAD: Pancreatic adenocarcinoma; TGCT: Testicular germ cell tumors; SKCM: Skin cutaneous melanoma; UVM: Uveal melanoma; BLCA: Bladder urothelial carcinoma; ACC: Adrenocortical carcinoma; KICH: Renal chromophobe carcinoma; CHOL: Cholangiocarcinoma.

Similarly, we examined the association between CCNB1 expression levels and additional pathological parameters, namely, T-stage, N-stage, and M-stage. Our analysis revealed a significant correlation between CCNB1 expression and T-stages in 11 cancer types, which included LUAD, BRCA, KIRP, KIPAN, PRAD, KIRC, LUSC, LIHC, PAAD, TGCT, and ACC (Figure 5A). For N-stages, a significant correlation with CCNB1 expression was observed in ten cancer types, namely, LUAD, COAD, COADREAD, BRCA, KIRP, KIPAN, PRAD, KIRC, LUSC, and ACC (Figure 5B). Furthermore, CCNB1 expression was significantly correlated with M-stages in six cancer types, specifically COADREAD, KIRP, KIPAN, KIRC, LUSC, and ACC (Figure 5C).

### Pan-cancer analysis of the prognostic value of CCNB1

**Figure 6.**
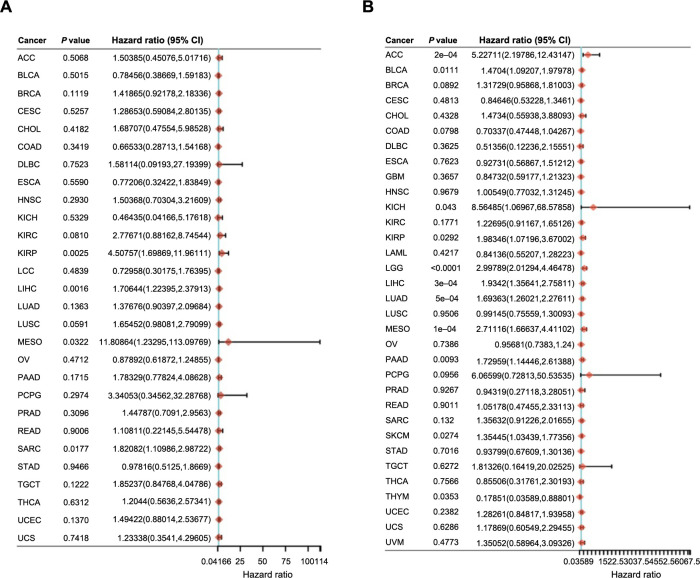
**Prognostic relevance of CCNB1 expression across various cancer types.** (A) Illustrating the Cox regression analysis of the relationship between CCNB1 expression and DFS across 28 cancer types; (B) Illustrating the Cox regression analysis of the relationship between CCNB1 expression and OS across 33 cancer types. CCNB1: Cyclin B1; DFS: Disease-free survival; OS: Overall survival; CI: Confidence interval; ACC: Adrenocortical carcinoma; BLCA: Bladder urothelial carcinoma; BRCA: Breast invasive carcinoma; CESC: Cervical squamous cell carcinoma; CHOL: Cholangiocarcinoma; COAD: Colon adenocarcinoma; DLBC: Lymphoid neoplasm diffuse large B cell lymphoma; ESCA: Esophageal carcinoma; GBM: Glioblastoma; HNSC: Head and neck squamous cell carcinoma; KICH: Renal chromophobe carcinoma; KIRC: Renal clear cell carcinoma; KIRP: Renal papillary cell carcinoma; LAML: Acute myeloid leukemia; LGG: Brain lower grade glioma; LIHC: Liver hepatocellular carcinoma; LUAD: Lung adenocarcinoma; LUSC: Lung squamous cell carcinoma; MESO: Mesothelioma; OV: Ovarian serous cystadenocarcinoma; PAAD: Pancreatic adenocarcinoma; PCPG: Pheochromocytoma and paraganglioma; PRAD: Prostate adenocarcinoma; READ: Rectum adenocarcinoma; SARC: Sarcoma; SKCM: Skin cutaneous melanoma; STAD: Stomach adenocarcinoma; TGCT: Testicular germ cell tumors; THCA: Thyroid carcinoma; THYM: Thymoma; UCEC: Uterine corpus endometrial carcinoma; UCS: Uterine carcinosarcoma; UVM: Uveal melanoma.

**Figure 7.**
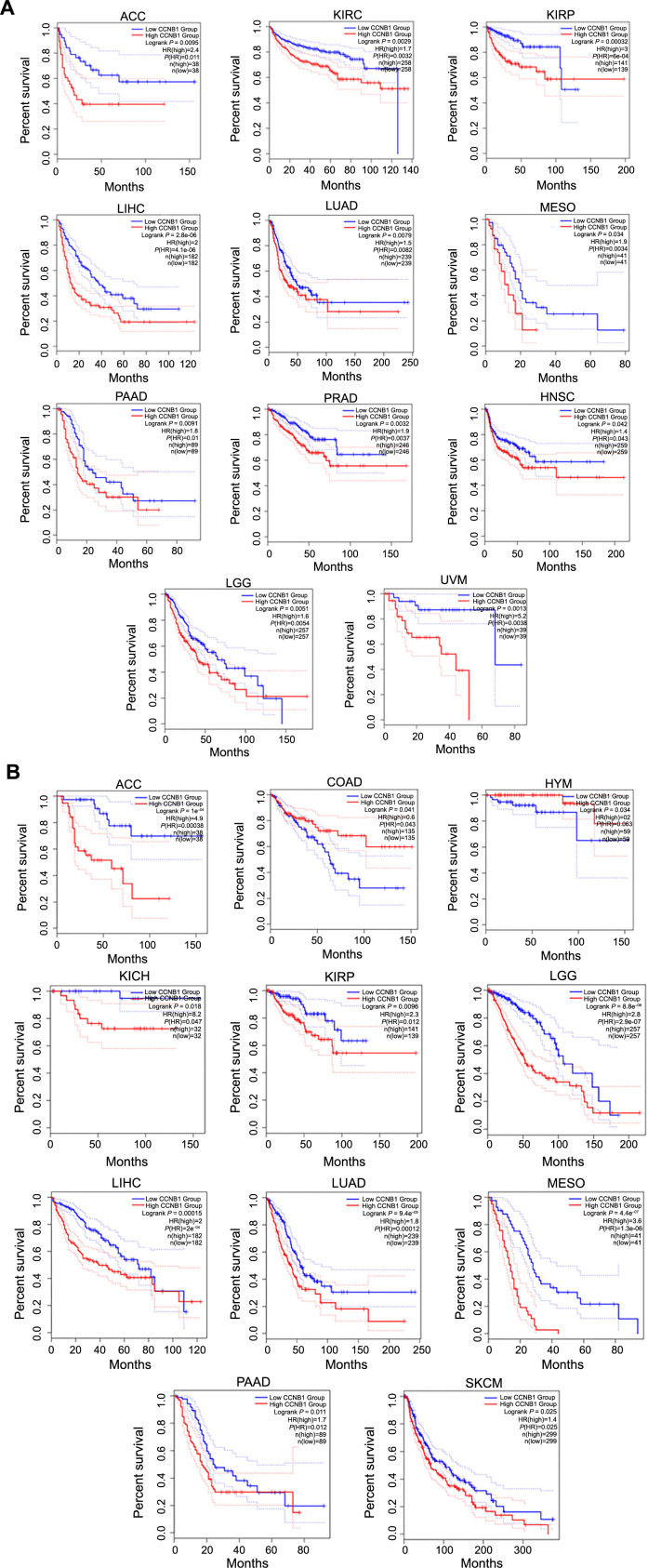
**Prognostic relevance of CCNB1 expression across various cancer types.** (A) Showcasing Kaplan–Meier plots depicting the association between CCNB1 expression and DFS in across various cancer types; (B) Showcasing Kaplan–Meier plots depicting the association between CCNB1 expression and OS across various cancer types. CCNB1: Cyclin B1; DFS: Disease-free survival; OS: Overall survival; CI: Confidence interval; ACC: Adrenocortical carcinoma; KIRC: Renal clear cell carcinoma; KIRP: Renal papillary cell carcinoma; LIHC: Liver hepatocellular carcinoma; LUAD: Lung adenocarcinoma; MESO: Mesothelioma; PAAD: Pancreatic adenocarcinoma; PRAD: Prostate adenocarcinoma; HNSC: Head and neck squamous cell carcinoma; LGG: Brain lower grade glioma; UVM: Uveal melanoma; COAD: Colon adenocarcinoma; THYM: Thymoma; KICH: Renal chromophobe carcinoma; SKCM: Skin cutaneous melanoma.

Based on the discovery of the CCNB1 expression levels in different tumor clinico-pathological stages, we further assessed the relationship between CCNB1 expression and patient prognosis across various cancers, using Cox regression and Kaplan–Meier analyses. The survival metrics primarily comprised of the DFS and the OS. The Cox regression analysis revealed that high CCNB1 expression significantly correlated with shorter DFS in four cancer types, namely, LIHC, KIRP, SARC, and MESO, out of the 28 cancer types evaluated (Figure 6A). Analysis of 33 cancer types suggested that high CCNB1 expression was significantly associated with reduced OS in 11 cancer types, including LGG, MESO, ACC, LIHC, LUAD, PAAD, BLCA, SKCM, KIRP, THYM, and KICH (Figure 6B). Furthermore, the Kaplan–Meier analysis revealed that elevated CCNB1 expression is associated with poor DFS in 11 cancer types, including ACC, KIRC, KIRP, LIHC, LUAD, MESO, PAAD, PRAD, HNSC, LGG, and UVM (Figure 7A), and poorer OS in nine types of cancer, namely, ACC, KICH, KIRP, LGG, LIHC, LUAD, MESO, PAAD, and SKCM. Conversely, higher CCNB1 expression correlated with improved OS in COAD and THYM (Figure 7B).

These findings indicated that CCNB1 expression inversely correlates with survival in certain cancers, reinforcing its potential as a prognostic biomarker.

### Pan-cancer analysis of the methylation level and genetic alteration of CCNB1

**Figure 8.**
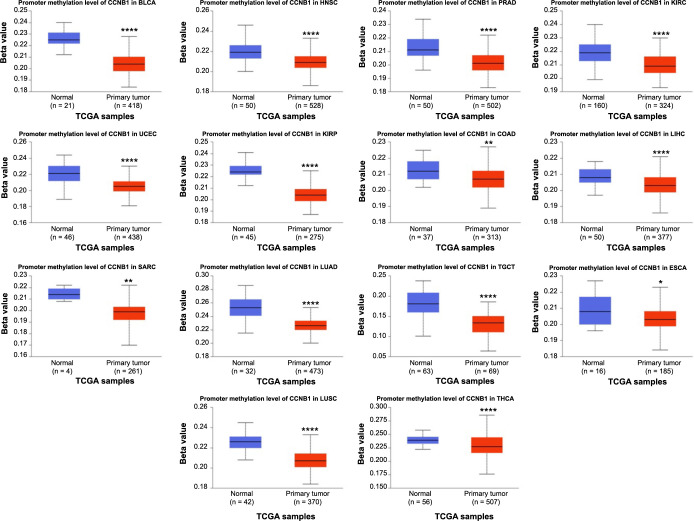
**CCNB1 promoter methylation levels across cancer types.** CCNB1: Cyclin B1; TCGA: The Cancer Genome Atlas; BLCA: BLCA: Bladder urothelial carcinoma; HNSC: Head and neck squamous cell carcinoma; PRAD: Prostate adenocarcinoma; KIRC: Renal clear cell carcinoma; UCEC: Uterine corpus endometrial carcinoma; KIRP: Renal papillary cell carcinoma; COAD: Colon adenocarcinoma; LIHC: Liver hepatocellular carcinoma; SARC: Sarcoma; LUAD: Lung adenocarcinoma; TGCT: Testicular germ cell tumors; ESCA: Esophageal carcinoma; LUSC: Lung squamous cell carcinoma; THCA: Thyroid carcinoma.

In the human genome, DNA methylation serves as an epigenetic mechanism that regulates gene expression [19]. It is implicated in gene transcription and frequently altered during carcinogenesis [19]. We assessed the DNA methylation status of CCNB1 using the UALCAN database and observed a significant decrease in methylation levels in 14 cancer types, including BLCA, HNSC, PRAD, KIRC, UCEC, KIRP, COAD, LIHC, SARC, LUAD, TGCT, ESCA, LUSC, and THCA, compared to their corresponding normal tissues (Figure 8). Recent research indicated that abnormal DNA methylation accumulation correlates positively with tumor progression [18]. The hypermethylation of specific tumor-suppressor genes can lead to their inactivation, while hypomethylation may contribute to cellular transformation [19]. Although the role of hypomethylation in cancerogenesis is less understood, some studies have proposed that it facilitates tumor formation and progression through various pathways, including its effects on transcription [20, 22]. Our findings demonstrated CCNB1 hypomethylation in specific cancers, suggesting a potential involvement in tumorigenesis by promoting cellular transformation. However, these findings warrant further experimental validation.

The progression of cancer is widely recognized to occur through a series of histopathological stages, with the current understanding attributing this progression to the accumulation of genetic alterations and subsequent changes in gene expression patterns [23].

**Figure 9.**
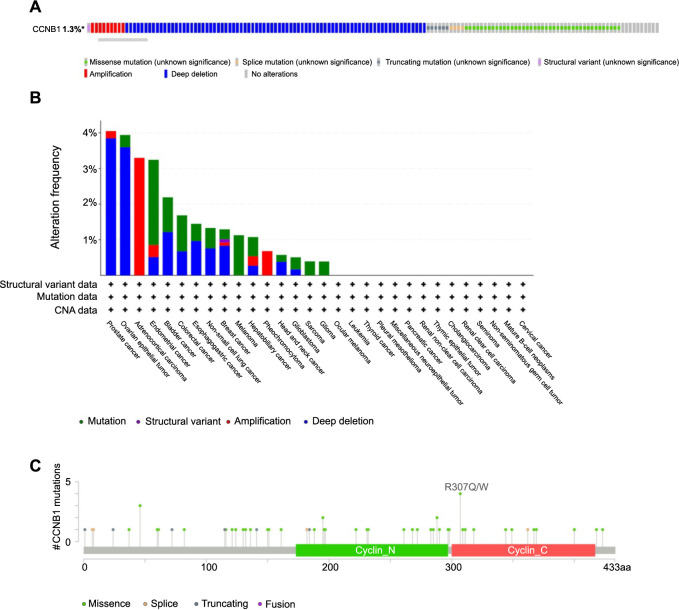
**Genetic alteration profile of CCNB1 in various cancers.** (A) Summarizing the total mutations in the CCNB1 gene as identified by the cBioPortal database; (B) Depicting the frequency of various CCNB1 gene alterations, classified by mutation type, as assessed by the cBioPortal database; (C) Illustrating a mutation diagram of CCNB1, detailing the specific mutation sites across different protein domains in various cancer types. CCNB1: Cyclin B1; CNA: Copy number alteration.

Analysis of CCNB1 genetic alterations was conducted using the cBioPortal database (TCGA, Pan-cancer Atlas). The results demonstrated that CCNB1 alterations were present in 1.3% (140/10950) of pan-cancer patients (Figure 9A), indicating that CCNB1 genetic alterations could play a role in promoting cancer progression. Further analysis of the mutation frequency of the CCNB1 gene across various cancer types showed that prostate cancer (4.05%), ovarian epithelial tumor (3.94%), ACC (3.3%), endometrial cancer (3.24%), and bladder cancer (2.19%) had the highest frequencies. Notably, deep deletion and mutation were the most common alteration types among the cancers studied (Figure 9B). To elucidate the mutation landscape of CCNB1 across protein domains in pan-cancer, we identified a total of 59 mutation sites ranging from amino acid positions 0–433 (Figure 9C).

### Pan-cancer analysis of the CCNB1 expression and immune cell infiltration

**Figure 10.**
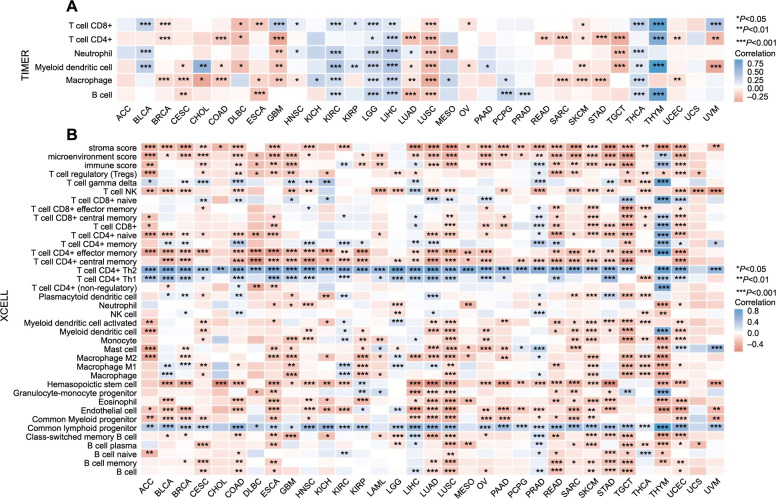
**Interplay between CCNB1 expression and immune cell infiltration across cancer types.** (A) Illustrating the relationship between CCNB1 expression and the immune cell infiltration in different cancers as analyzed by the TIMER algorithm; (B) Showcasing the relationship between CCNB1 expression and the immune cell infiltration in different cancers as analyzed by the xCELL algorithm. CCNB1: Cyclin B1; TIMER: Tumor Immune Estimation Resource; CD: Cluster of differentiation; ACC: Adrenocortical carcinoma; BLCA: Bladder urothelial carcinoma; BRCA: Breast invasive carcinoma; CESC: Cervical squamous cell carcinoma; CHOL: Cholangiocarcinoma; COAD: Colon adenocarcinoma; DLBC: Lymphoid neoplasm diffuse large B cell lymphoma; ESCA: Esophageal carcinoma; GBM: Glioblastoma; HNSC: Head and neck squamous cell carcinoma; KICH: Renal chromophobe carcinoma; KIRC: Renal clear cell carcinoma; KIRP: Renal papillary cell carcinoma; LAML: Acute myeloid leukemia; LGG: Brain lower grade glioma; LIHC: Liver hepatocellular carcinoma; LUAD: Lung adenocarcinoma; LUSC: Lung squamous cell carcinoma; MESO: Mesothelioma; OV: Ovarian serous cystadenocarcinoma; PAAD: Pancreatic adenocarcinoma; PCPG: Pheochromocytoma and paraganglioma; PRAD: Prostate adenocarcinoma; READ: Rectum adenocarcinoma; SARC: Sarcoma; SKCM: Skin cutaneous melanoma; STAD: Stomach adenocarcinoma; TGCT: Testicular germ cell tumors; THCA: Thyroid carcinoma; THYM: Thymoma; UCEC: Uterine corpus endometrial carcinoma; UCS: Uterine carcinosarcoma; UVM: Uveal melanoma; NK cells: Natural killer cells.

Solid tumors are intricate structures composed of neoplastic cells intermingled with various normal cell types, including immune cells. These cells persistently interact, influencing tumor growth and immune function. In particular, immune cells can both inhibit and promote tumor proliferation and are integral in regulating anti-tumor immune responses [24]. Several studies have indicated that CCNB1 may affect the pattern of immune infiltration in certain cancers [16, 25]. Therefore, we examined the relationship between CCNB1 expression and immune infiltration in pan-cancer settings. Utilizing the TIMER analysis, we observed a significant association between CCNB1 expression and the abundance of several types of infiltrating immune cells: CD8+ T cells in 16 cancers, CD4+ T cells in 16 cancers, neutrophils in 11 cancers, myeloid dendritic cells in 19 cancers, macrophages in 20 cancers, and B cells in 11 cancers (Figure 10A).

Further analysis using the xCELL algorithm investigated the relationship between CCNB1 expression levels and the infiltration of different immune cell subtypes. Among 38 immune cell subtypes, significant negative correlations with CCNB1 expression were found in cancers such as ACC, BLCA, BRCA, ESCA, GBM, HNSC, LUAD, LUSC, READ, SARC, SKMC, STAD, TGCT, and UCEC. Conversely, a significant positive correlation was observed in LIHC, PRAD, and THYM. Most notably, CCNB1 expression demonstrated the strongest positive correlation with CD4+ Th2 T cell, CD4+ Th1 T cell, and common lymphoid progenitor cells across various cancer types (Figure 10B).

These findings suggested a potential association between CCNB1 and the recruitment of diverse immune cells within the tumor microenvironment.

### Pan-cancer analysis of the correlation between CCNB1 expression and immune checkpoint genes, TMB, and MSI

**Figure 11.**
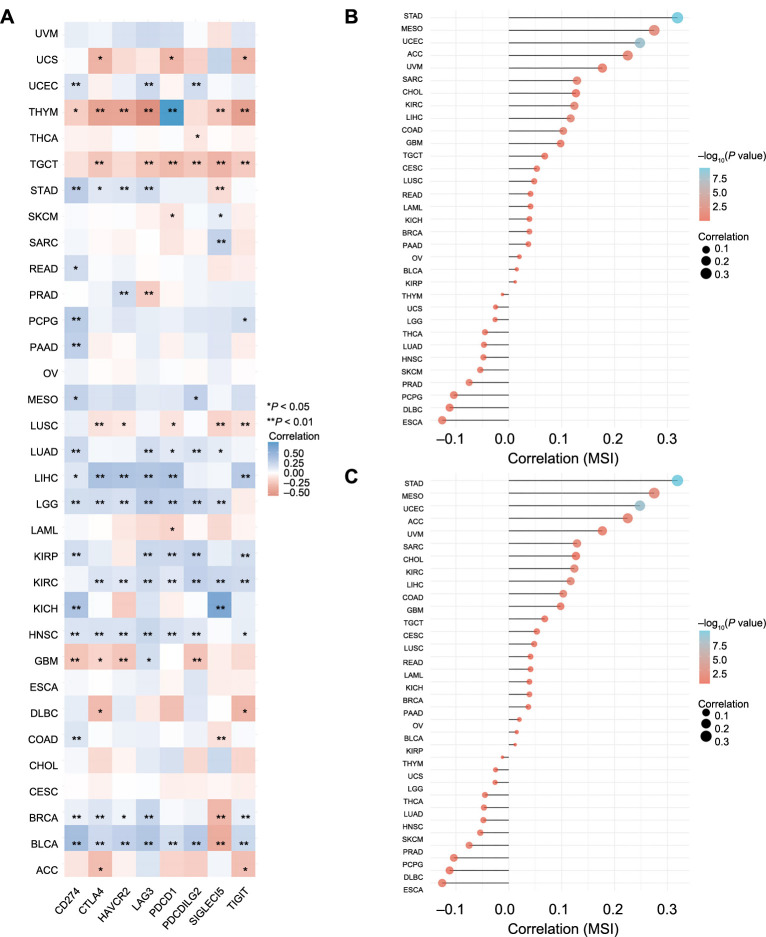
**Correlations between CCNB1 expression, immune checkpoints, and genomic biomarkers in pan-cancers.** (A) Displaying the relationship between CCNB1 expression and various immune checkpoint genes across various cancers; (B) Showcasing the relationship between CCNB1 expression and TMB across various cancers; (C) Illustrating the relationship between CCNB1 expression and MSI across various cancers. CCNB1: Cyclin B1; TMB: Tumor mutational burden; MSI: Microsatellite instability; ACC: Adrenocortical carcinoma; BLCA: Bladder urothelial carcinoma; BRCA: Breast invasive carcinoma; CESC: Cervical squamous cell carcinoma; CHOL: Cholangiocarcinoma; COAD: Colon adenocarcinoma; DLBC: Lymphoid neoplasm diffuse large B cell lymphoma; ESCA: Esophageal carcinoma; GBM: Glioblastoma; HNSC: Head and neck squamous cell carcinoma; KICH: Renal chromophobe carcinoma; KIRC: Renal clear cell carcinoma; KIRP: Renal papillary cell carcinoma; LAML: Acute myeloid leukemia; LGG: Brain lower grade glioma; LIHC: Liver hepatocellular carcinoma; LUAD: Lung adenocarcinoma; LUSC: Lung squamous cell carcinoma; MESO: Mesothelioma; OV: Ovarian serous cystadenocarcinoma; PAAD: Pancreatic adenocarcinoma; PCPG: Pheochromocytoma and paraganglioma; PRAD: Prostate adenocarcinoma; READ: Rectum adenocarcinoma; SARC: Sarcoma; SKCM: Skin cutaneous melanoma; STAD: Stomach adenocarcinoma; TGCT: Testicular germ cell tumors; THCA: Thyroid carcinoma; THYM: Thymoma; UCEC: Uterine corpus endometrial carcinoma; UCS: Uterine carcinosarcoma; UVM: Uveal melanoma; *CD274*: Cluster of differentiation 274; *CTLA4*: Cytotoxic T-lymphocyte-associated protein 4; *HAVCR2*: Hepatitis A virus cellular receptor 2; *LAG3*: Lymphocyte-activation gene 3; *PDCD1*: Programmed cell death protein 1; *PDCD1LG2*: Programmed cell death 1 ligand 2; *SIGLEC15*: Sialic acid binding Ig-like lectin 15; *TIGIT*: T cell immunoreceptor with Ig and ITIM domains.

In recent years, there has been significant advancement in the field of immunotherapy, which is now widely acknowledged as an effective approach for managing cancer progression [26]. With the observed correlation between CCNB1 expression and immune cell infiltration, we delved into the relationship between CCNB1 expression and immune checkpoint genes. Our research revealed a positive correlation between these genes and several cancers, including UCEC, STAD, LUAD, LIHC, LGG, KIRP, KIRC, KICH, HNSC, BRCA, and BLCA. However, a negative association was observed in THYM, TGCT, LUSC, and GBM (Figure 11A).

Genomic biomarkers such as MSI testing and TMB have been utilized for the identification of patients who are most likely to derive benefit from immunotherapy [27]. Our investigation into the relationship between CCNB1 expression and TMB indicated a significant correlation in cancers, namely, STAD, ACC, CHOL, LGG, DLBC, LUAD, BRCA, PAAD, KICH, SARC, PRAD, USC, LUSC, SKCM, COAD, UCEC, BLCA, LAML, KIRC, HNSC, THCA, and THYM (Figure 11B). Subsequently, we examined the association between CCNB1 expression and MSI across various cancers, uncovering positive correlations in STAD, MESO, UCEC, ACC, SARC, KIRC, LIHC, and COAD (Figure 11C).

These findings underscored the association of CCNB1 with immune checkpoint genes, TMB, and MSI. A comprehensive investigation into the molecular interactions of these factors may potentially lead to the discovery of novel immunotherapy strategies.

### Enrichment analysis of CCNB1 in pan-cancers

**Figure 12.**
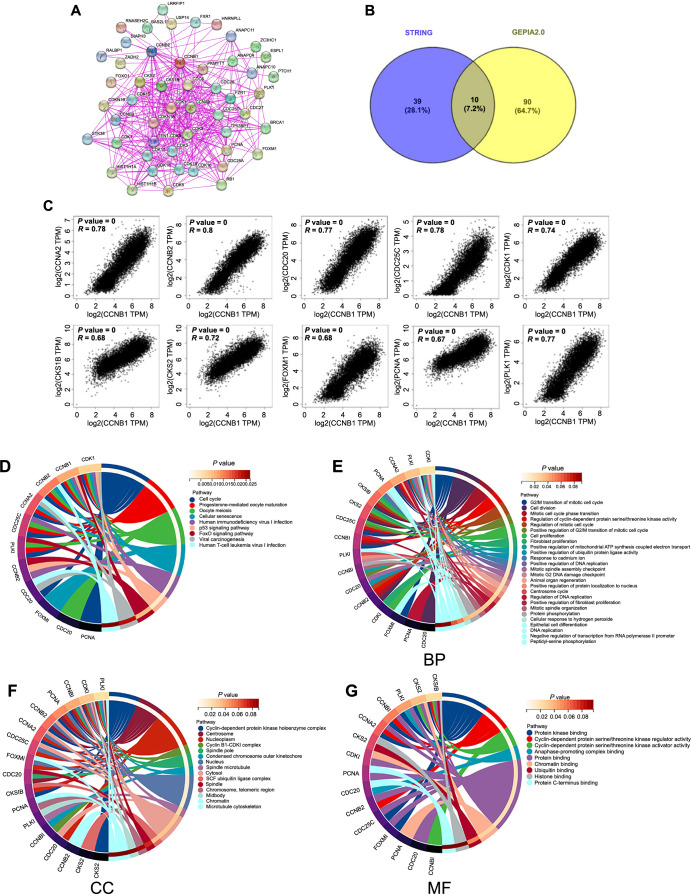
**Comprehensive enrichment analysis of CCNB1 in pan-cancers.** (A) Displaying the network of potential CCNB1-binding proteins as identified by STRING database analysis; (B) A Venn diagram illustrating the intersection between CCNB1-binding genes and CCNB1-related genes; (C) Demonstrating significant positive correlations between CCNB1 and 10 potential CCNB1-binding genes; (D) Summarizing the KEGG pathway analysis results for CCNB1 and the 10 CCNB1-ralated genes; (E–G) Depicting the GO enrichment analysis results for CCNB1 and the 10 associated genes, categorized into BP (E), CC (F), and MF (G). CCNB1: Cyclin B1; STRING: Search Tool for the Retrieval of Interacting Genes/Proteins; KEGG: Kyoto Encyclopedia of Genes and Genomes; GO: Gene Ontology; BP: Biological process; CC: Cellular component; MF: Molecular function; GEPIA: Gene Expression Profiling Interactive Analysis; TPM: Transcripts per million; *CCNA2*: Cyclin A2; *CCNB2*: Cyclin B2; *CDC20*: Cell division cycle 20 gene; *CDC25C*: Cell division cycle 25C gene; *CDK1*: Cyclin-dependent kinase 1; *CKS1B*: CDC28 Protein kinase regulatory subunit 1B; *CKS2*: CDC28 Protein kinase regulatory subunit 2; *FOXM1*: Forkhead box M1; *PCNA*: Proliferating cell nuclear antigen; *PLK1*: Polo-like kinase 1; FoxO: Forkhead box O; ATP: Adenosine triphosphate; SCF: SKP1-CUL1-F-box protein.

To investigate the molecular mechanisms underlying CCNB1’s role in tumor progression, we examined its PPI network, analyzed genes associated with CCNB1, and conducted enrichment analysis. Using the STRING database, we identified 50 potential proteins that interact with CCNB1 (Figure 12A). Further, the GEPIA 2.0 web tool facilitated the identification of the top 100 genes associated with CCNB1 across all cancer tissues from the TCGA database. A Venn diagram approach yielded ten CCNB1-associated genes common to both datasets (Figure 12B), namely, cyclin B2 (*CCNB2*), cyclin A2 (*CCNA2*), cell division cycle 25C gene (*CDC25C*), Polo-like kinase 1 (*PLK1*), cell division cycle 20 gene (*CDC20*), *CDK1*, CDC28 protein kinase regulatory subunit 1B (*CKS1B*), proliferating cell nuclear antigen (*PCNA*), forkhead box M1 (*FOXM1*), and CDC28 protein kinase regulatory subunit 2 (*CKS2*), all exhibiting a positive correlation with CCNB1 (Figure 12C). Additionally, we integrated the genes associated with CCNB1 from both STRING and GEPIA databases for a comprehensive KEGG pathway and GO terms enrichment analysis. The KEGG pathway analysis highlighted an enrichment of these genes in pathways including “Cell cycle,” “Cellular senescence,” “Forkhead box O (FoxO) signaling pathway,” and “p53 signaling pathway” (Figure 12D). GO enrichment analysis across BPs, Cellular components (CCs), and Molecular functions (MFs) categories indicated CCNB1’s predominant involvement in cell division processes (Figure 12E–12G).

## Discussion

Cancer presents a complex assortment of diseases that account for 7.5 million fatalities annually [28]. Despite extensive research aimed at understanding the mechanisms underlying various cancers and developing effective treatments, significant advances in clinical therapies have been met with relatively limited success [28]. However, recently, a great number of researchers suggest that the genomic revolution may break the impasse [28]. TCGA launched the Pan-Cancer Program during a conference in Santa Cruz, California, on October 26 2012. The program is supposed to investigate the commonalities, differences, and new topics across various tumor types and tissue origins [29]. An increasing body of pan-cancer analyses has been shedding light on the relationship between genetic abnormalities and the initiation and development of cancer.

CCNB1, a member of the cyclin family, is essential for the cell cycle regulatory machinery [30]. Current research has implicated CCNB1 in the pathogenesis of various cancers, such as gastric, pancreatic, and cervical squamous cell carcinomas [13, 30–33]. For instance, Fang et al. [9] demonstrated an association between aberrant CCNB1 expression and lower survival rates in breast cancer. In ovarian cancer, the CCNB1 expression was markedly increased and has been linked to enhanced proliferation, migration, and invasion of tumor cells [34]. Similarly, Zhang et al. [33] found that CCNB1 silencing could inhibit cell proliferation and promote senescence in pancreatic cancer. The expression of CCNB1 is regulated by a variety of molecular mechanisms that differ across cancer types [35–37]. The abnormal CCNB1 expression is not only affecting tumor cell proliferation but also apoptosis, migration, and invasion [38–40]. Additionally, recent studies have revealed a correlation between CCNB1 and immune infiltration. For example, CCNB1 overexpression was shown to promote immune infiltration in hepatocellular carcinoma [16]. To the best of our knowledge, there are no published reports on a comprehensive pan-cancer analysis of CCNB1. Therefore, our study aims to elucidate the role of CCNB1 across a spectrum of human cancers using bioinformatics methods.

As highlighted earlier, CCNB1 upregulation has been linked to tumor progression in several cancer types. This study represents the first comprehensive pan-cancer investigation of CCNB1 expression, comparing it to corresponding normal tissues. What captured our interest was CCNB1’s remarkable detection performance. Within the GEPIA database, CCNB1 mRNA levels were significantly higher in 22 types of cancer, while the Sangerbox database showed significant upregulation in 32 cancer types. A comparative analysis of the two sets revealed increased CCNB1 mRNA expression in 28 cancers, namely, ACC, BLCA, BRCA, CESC, COAD, ESCA, GBM, HNSC, LIHC, LUAD, LUSC, OV, PAAD, PRAD, READ, SKCM, STAD, TGCT, UCEC, DLBC, THYM, GBMLGG, STES, KIPAN, COADREAD, WT, ALL, and UCS. Considering the role of post-transcriptional regulation, we did not solely rely on mRNA levels for our analysis. Consequently, we assessed CCNB1 protein levels and discovered significant upregulation in nine cancer types. Excitingly, in these cancers, CCNB1 protein expression aligned with the mRNA expression trends. However, protein expression data for certain cancer types were unavailable in the UALCAN database. Analysis from the HPA database indicated that CCNB1 protein expression was elevated in 12 cancers, consistent with the mRNA expression profiles in these cancer types. Our findings suggest that the dysregulation of CCNB1 is involved in tumor progression across multiple cancer types. Nonetheless, these observed differences warrant confirmation with sufficient experimental data.

Analysis of the relationship between CCNB1 expression levels and cancer clinico-pathological stages indicated that abnormal CCNB1 expression could serve as a prognostic indicator in cancer patients. Our research revealed an association between CCNB1 upregulation and progression through clinical stages across various cancers. The logistic regression analysis has confirmed the clinical utility of CCNB1 in differentiating between early and late clinical stages, particularly in ACC and KIRP. In addition, we observed that an increase in CCNB1 expression correlates with a higher histological grade in ten types of cancer. Furthermore, elevated CCNB1 expression levels were significantly linked to the progression of TNM stages in several cancer types. Remarkably, KIPAN and KIRC consistently appeared in significant findings across these five parameters. These findings suggest that CCNB1 overexpression may contribute to the progression of clinico-pathological stages in certain cancers. This discovery could potentially enhance the clinical significance of CCNB1 as a novel pathological staging marker for a range of cancer types. However, further research is necessary to elucidate the specific mechanisms involved. Our results also reinforce the potential of CCNB1 as a valuable prognostic biomarker.

To gain a deeper understanding of CCNB1’s prognostic value, we conducted survival analyses. Both Cox and Kaplan–Meier survival analyses indicated that higher CCNB1 expression correlates significantly with poorer OS in patients across 11 cancer types, namely, LGG, MESO, ACC, LIHC, LUAD, PAAD, BLCA, SKMC, KIRP, THYM, and KICH. Similarly, these analyses revealed that elevated CCNB1 levels are significantly correlated with worse DFS in 12 cancer types, including SARC, ACC, KIRC, KIRP, LIHC, LUAD, MESO, PAAD, PRAD, HNSC, LGG, and UVM. These results further support the potential of CCNB1 as an independent prognostic factor in various cancers.

Epigenetic mechanisms play a crucial role in the development of multicellular organisms, facilitating precise temporal and spatial regulation of gene expression [41]. Dysregulation of DNA methylation has been closely linked to the onset of various diseases, including cancer [42]. Extensive research has shown that aberrant DNA methylation correlates with poor cancer prognosis [43–46]. Our study showed that DNA methylation of CCNB1 was downregulated in 14 cancer types. Hypomethylation can lead to cell transformation, whereas hypermethylation of certain tumor-suppressor genes often results in gene inactivation [19]. To the best of our knowledge, the mechanisms by which hypomethylation contributes to carcinogenesis are not well understood. Some studies indicate that DNA hypomethylation associated with cancer likely facilitates tumor formation and progression through various pathways [20]. For example, one study confirmed that DNA hypomethylation enhances gene transcription and tumor proliferation [19]. As a cell cycle regulator, CCNB1 may promote tumor cell proliferation via DNA hypomethylation. Furthermore, researchers have suggested that analyzing DNA methylation patterns in circulating tumor DNA could significantly advance minimally invasive cancer detection and classification [47]. Thus, investigating the methylation mechanism of CCNB1 could be clinically beneficial. Our findings were significant, suggesting that hypomethylation of CCNB1 may promote tumorigenesis and tumor progression.

Mutations, which include deletions, additions, amplifications, and recombinations, are changes in the DNA nucleotide sequence [48]. Over the past decade, studies have shown that gene mutations can facilitate or “drive” tumor development [49]. The acquisition of genetic mutations is a primary mechanism responsible for the dysregulation of cell proliferation, invasion, and apoptosis, all critical processes in oncogenesis [50]. Our data exhibited that CCNB1 has a mutation rate of 1.3% in pan-cancer. Deep deletions and mutations were the most prevalent types observed. The mutation frequency of the CCNB1 gene was comparatively high in prostate cancer, ovarian epithelial tumor, ACC, endometrial cancer, and bladder cancer, highlighting the need to investigate the link between CCNB1 mutations and urogenital system cancers. In these five types, CCNB1 expression was elevated compared to their matched normal tissues, suggesting that mutations may lead to CCNB1 dysregulation. Consequently, CCNB1 is likely implicated in tumor progression. Many devastating human diseases are caused by individual genetic mutations that prevent somatic cells from performing their essential functions during cell transformation. As such, targeted gene therapies have emerged as a promising approach to treat these conditions [51]. Our findings indicate that CCNB1 could be a potential therapeutic target.

Our study is the first to demonstrate a potential association between CCNB1 mRNA expression and immune infiltration across various cancers. We observed that CCNB1 expression significantly correlates with the infiltration levels of CD8+ T-cells, CD4+ T-cells, macrophages, neutrophils, myeloid dendritic cells, and B cells in many cancer types. Additionally, there appears to be a specific relationship with certain immune cell subtypes. These findings suggested that CCNB1 plays an important role in the tumor immune microenvironment. Predominantly, the expression of CCNB1 was inversely correlated with the level of immune cell infiltration, indicating a potential relationship between CCNB1 gene expression and tumor immune cell infiltration, although the exact molecular mechanisms underlying this relationship remain to be elucidated.

We also assessed the relationship between the *CCNB1* gene and immune checkpoint genes in pan-cancer. Notably, there was a positive correlation between CCNB1 expression and most immune checkpoint genes in many cancers, including UCEC, STAD, LUAD, LIHC, LGG, KIRP, KIRC, KICH, HNSC, BRCA, and BLCA. However, a few tumors, such as THYM and TGCT, showed an inverse relationship with these genes.

Building on these findings, we further analyzed the relationship between CCNB1 and two key genomic biomarkers, TMB and MSI. We found that CCNB1 expression levels were significantly associated with TMB in a wide range of cancers, including STAD, ACC, CHOL, LGG, DLBC, LUAD, BRCA, PAAD, KICH, SARC, PRAD, USC, LUSC, SKCM, COAD, UCEC, BLCA, LAML, KIRC, HNSC, THCA, and THYM. Additionally, CCNB1 expression correlated with MSI in cancers such as STAD, MESO, UCEC, ACC, SARC, KIRC, LIHC, and COAD. In summary, CCNB1 is associated with immune checkpoint genes, TMB, and MSI, highlighting its potential as a promising target for immunotherapy. Furthermore, CCNB1 could serve as a novel biomarker for distinguishing the MSI-Low from the microsatellite stable (MSS) and MSI-High groups.

We finally explored potential binding proteins and genes associated with CCNB1 expression across the different cancer types. By combining data from GEPIA and STRING databases, we identified ten common genes, namely, *CCNB2*, *CCNA2*, *CDC25C*, *PLK1*, *CDC20*, *CDK1*, *CKS1B*, *FOXM1*, *PCNA*, and *CKS2*, that were strongly positively correlated with CCNB1. The results of the GO and KEGG enrichment analyses conducted with these genes using both databases revealed significant enrichment of CCNB1 in pathways related to the “Cell cycle,” “Cellular senescence,” “p53 signaling pathway,” and “FoxO signaling pathway.” These findings suggested that CCNB1 plays a crucial role in the development and progression of cancer by regulating cell cycle processes. The p53 signaling pathway, which functions as a complex cellular stress response network and a tumor suppressor pathway, involves the tumor protein 53 (*TP53*) gene, the gene most commonly mutated in human cancer [52]. The FoxO transcription factors belong to the forkhead family and play crucial roles in cell fate decisions. This subfamily serves a pivotal function as a tumor suppressor across a broad spectrum of cancers [53]. CCNB1 may facilitate oncogenesis by influencing the p53 and FoxO signaling pathways, both of which have been proven to participate in tumorigenesis across most cancer types. Indeed, several studies have validated this relationship.

## Conclusion

In summary, our research revealed widespread overexpression of CCNB1 across various cancer types and demonstrated its correlation with clinical prognosis. Our findings suggest that CCNB1 has the potential to serve as an independent prognostic biomarker for numerous cancers, with its expression level varying across different cancer types. The expression of CCNB1 is associated with clinicopathological stages, prognosis, immune cell infiltration, and immune-related genes in most cancer types. DNA hypomethylation and gene mutation of CCNB1 may contribute to oncogenesis and tumor progression. CCNB1 plays a crucial role in tumor immunology and holds significant prognostic value, presenting potential as a new biomarker for both prognosis and immunotherapy across various cancers. However, our results were based on bioinformatics analysis and preliminary functional exploration, without experimental validation. Future in vitro or in vivo experiments are necessary to validate the practical applications of CCNB1. Additionally, a detailed study of the specific molecular mechanisms underlying CCNB1 overexpression is essential. This research could provide valuable insights into developing new therapeutic strategies for treating cancer.
